# Maternal and Perinatal Factors Associated with the Human Milk Microbiome

**DOI:** 10.1093/cdn/nzaa027

**Published:** 2020-03-09

**Authors:** Hans Demmelmair, Esther Jiménez, Maria Carmen Collado, Seppo Salminen, Michelle K McGuire

**Affiliations:** 1 Dr. von Hauner Children´s Hospital, University of Munich Medical Center, Munich, Germany; 2 ProbiSearch SLU, Madrid, Spain; 3 Department of Nutrition, Food Science, and Technology, University Complutense, Madrid, Spain; 4 Institute of Agrochemistry and Food Technology, Spanish National Research Council, Valencia, Spain; 5 Functional Foods Forum, University of Turku, Turku, Finland; 6 Margaret Ritchie School of Family and Consumer Sciences, University of Idaho, Moscow, ID, USA

**Keywords:** milk microbiome, variation, human milk, composition, entero-mammary pathway, geographical setting, mastitis

## Abstract

Microbes are present in human milk regardless of the mother's health. The origins of the milk microbiota likely include the mother's skin, infant's mouth, and transfer from the maternal gastrointestinal (GI) tract. Prominent bacterial taxa in human milk are *Staphylococcus* and *Streptococcus*, but many other genera are also found including anaerobic *Lactobacillus*, *Bifidobacterium*, and *Bacteroides*. The milk microbiome is highly variable and potentially influenced by geographic location, delivery mode, time postpartum, feeding mode, social networks, environment, maternal diet, and milk composition. Mastitis alters the milk microbiome, and the intake of Lactobacilli has shown potential for mastitis treatment and prevention. Although milk and infant fecal microbiomes are different, their variations appear to be related – suggesting that milk is an important contributor of early GI colonization. Nonetheless, nothing is known regarding whether the milk microbiome influences infant health. Further research and clinical interventions are needed to determine if changes in the microbiomes of human milk and infant formula/food impact health.

## Microbiome and Early Development

The maternal microbiome has long been recognized as one of the essential factors determining the neonatal microbiome. In addition to skin contact and exposure to vaginal secretions and fecal materials during birth as possible sources of bacteria for the infant, human milk may be one of the important links between maternal and infant microbiomes. Indeed, it is possible that exposure of the infant to the microbiome inherent in the milk produced by his/her mother influences development of the infant's gastrointestinal (GI) microbiome, respiratory tract microbiome, immune system (including the development of tolerance), and additional health outcomes (1–3).

Human milk is the “gold standard” for infant nutrition with many epidemiologically demonstrated advantages for the infant, including decreased risk of respiratory tract infections, atopic dermatitis, asthma, obesity, type 1 and type 2 diabetes, necrotizing enterocolitis (NEC), gastroenteritis, and sudden infant death syndrome (4, 5). In addition to proteins, peptides, lipids, micronutrients, nucleotides, hormones, growth factors, immunomodulatory agents, living cells, and human milk oligosaccharides (HMO), microbes are now thought to be important, biologically active components of human milk (6, 7). Along with systemic effects, these milk components are thought to primarily target intestinal epithelia, and thus affect absorption of nutrients, mucosal permeability, cellular proliferation, GI bacteria, induction of superficial molecules, and regulation of cytokine production. In addition, microbes may have a systemic effect on the enteric nervous system and mucosal immune system (8).

Based on the demonstrated advantages of being breastfed (or fed human milk by other means), the microbiome of the healthy, vaginally delivered infant can be considered the “reference standard,” generally providing the best chance for optimal development of the offspring's immune system and metabolism. The infant GI microbiome develops during the first years of life towards an adult-like microbiome, a process that involves an increase in diversity (9, 10). Nutrition has a major impact on early infant GI microbiota composition and function. Although not confirmed in all studies (11), higher relative amounts of Bifidobacteria in feces are considered typical for human milk-fed infants, whereas higher relative abundances of Enterococci and Clostridia seem associated with formula feeding (12). Cessation of breastfeeding, rather than the introduction of other foods, seems to be required for maturation into an adult-like GI microbiome (13).

The infant's GI microbiome is extensively linked with the immune system, and understanding what is a “normal, healthy” infant GI microbiome in a distinct population could provide opportunities to improve the health of both breastfed and formula-fed infants in that population. This requires indepth understanding of the basic principles, including the microbial ecosystems during the perinatal period and its associations with human milk composition including the human milk microbiome. In addition, individual geographic requirements may exist. It is possible that what is normal and healthy in terms of the neonatal GI microbiome in one region and situation might be different from what is optimal in another region and culture. This shift in what is considered healthy based on environmental, cultural, and possibly evolutionary factors has been referred to as *eco-homeorhesis* (14) and may explain, at least in part, variation in many physiologic parameters (including both milk and fecal microbiomes) around the globe.

Although researchers and clinicians have long been interested in the microbial communities residing in the infant's GI tract, the ubiquitous presence of human milk-resident microbes has only recently been fully recognized. Indeed, milk was long considered sterile unless produced by an infected mammary gland or contaminated after it was expressed. Nonetheless, it is now generally accepted that human milk is a constant source of a variety of microbes to the infant (15). Since these microbes and their antigens are some of the first experienced by the immature infant's immune system, it is likely that they play an important role in an infant's immune development, including factors related to establishing tolerance to generally nonpathogenic microbes common in the infant's environment.

## Human Milk Microbiome

Human milk microbes have been studied by several groups using culture-dependent and culture-independent techniques. Culture-dependent techniques quantify cultivable bacteria and enable further study of the clones, whereas molecular techniques enable a more comprehensive description of bacterial diversity (16). With a variety of media, culture-dependent techniques have enabled the detection of *Staphylococcus*, *Streptococcus*, *Enterococcus*, and *Lactobacillus* species (all in the Firmicutes phylum); *Propionibacterium* and *Rothia* species (Actinobacteria phylum); and occasionally *Enterobacteriaceae* species (Proteobacteria phylum) in human milk (17–19). Although aerobic probiotic bacteria have been evaluated extensively, anaerobic *Bifidobacterium* species (Actinobacteria phylum) and *Bacteroides* species (Bacteroidetes phylum) have also been identified using culture-dependent techniques (18–20). Culture-independent methods have identified and quantified DNA of anaerobic bacteria not previously detected in human milk using culture-dependent techniques, including additional species of *Bacteroides* (Bacteroidetes phylum), *Clostridium* (Firmicutes phylum), *Eubacterium* (Firmicutes phylum), and *Veillonella* (Firmicutes phylum) (21).

In a recent systematic review, and considering only culture-independent identified bacterial genera, *Streptococcus* and *Staphylococcus* were identified as the predominant genera in milk produced by healthy women (22). Salminen and coworkers suggested that the role of early infant GI colonization by the genera *Lactobacillus* and *Staphylococcus* is related to the hygiene hypothesis (23). This hypothesis posits that changes of microbial exposure in early life due to a variety of factors, including improved hygiene and increased use of antibiotics, lead to differences in the immunological adjustment of infants to extra uterine life, which may be associated with immune dysfunction and increased inflammatory diseases (24). Human milk components (e.g. HMO, secretory IgA, lactoferrin) support a healthy early GI colonization and neonatal immune-system development. The human milk microbiome could also contribute to this process or directly interact with the neonatal GI tract, but processes are far from understood (25).

*Streptococcus* and *Staphylococcus* were both considered part of the core human milk microbiome in previous studies, and it is possible that both genera are universally present in the human milk microbiota, independent of geographic location or analytical technique applied (26, 27). This finding is supported by recently published data from the Canadian Healthy Infant Longitudinal Development (CHILD) cohort, which evaluated human milk samples produced by 393 mainly Caucasian mothers; overall, the most abundant taxa were identified as variants of *Streptococcus* (16%) and the third most abundant were variants of *Staphylococcus* (5%) (28). Further support that *Streptococcus* and *Staphylococcus* are found in human milk from very different settings, and may be considered typical for all human milk, comes from the INSPIRE project (Evolutionary and Sociocultural Aspects of Human Milk Composition). In this study, milk samples produced by 394 women were analyzed for their bacterial community structures using 16S methodologies (29). The investigators took care to standardize sample collection and storage in this crosscultural study including women from 6 African populations (rural and urban Gambia and Ethiopia, Kenya, Ghana), 2 European countries (Sweden, Spain), the USA (California, Washington/Idaho), and Peru. *Staphylococcus* was found in 99%, *Streptococcus* in 98%, and *Propionibacterium* in 76% of the milk samples, indicating the presence of a human milk core microbiome. However, there was substantial variation among cohorts; for instance, *Rhizobium* was the most abundant taxon in milk produced by women living in rural Ethiopia. Bacterial α-diversity also varied among cohorts. Although there were only limited associations between individual genera in milk and feces, community-level analyses suggested strong, positive associations between the complex communities in these sample types (29).

Important steps to increase knowledge about the role of the human milk microbiome in relation to infant health are to: *1*) identify the origin of human milk bacteria, *2*) understand the factors influencing the milk microbiome, and *3*) describe the importance of human milk and mammary bacteria in specific clinical situations such as preterm delivery, diarrhea, or mastitis. Increased mechanistic understanding may provide solutions to compensate for suboptimal clinical situations through supplementing breastfeeding mothers or by improving infant formulas through the addition of prebiotics or probiotics.

This critical review is based on a workshop, held in October 2017, to discuss these questions and the legal requirements to transfer such research findings into practice. For the review, the contribution of the experts to the workshop was combined with literature identified in the Web of Science applying a kind of snowball search and considered relevant for the topic. For the literature search no predefined inclusion or rejection criteria were applied, but the focus was put on observational and interventional studies in humans and no publications available after August 2019 were considered.

## Origin of Human Milk Bacteria

The presence of bacteria in human milk does not suggest a compromised health status of the lactating mother, but that bacteria are obligate components of this complex fluid that provides sole-source-nutrition (and other critical nonnutritive substances) to the neonate. Nevertheless, the presence of bacteria alone does not prove that they are beneficial for mother or infant. Understanding the sources and pathways of bacteria into the milk may give indications about their possible benefits.

It was initially assumed that bacteria in the milk of healthy mothers were contaminants from maternal skin. This agrees with the observation that Streptococci, Corynebacteria, and Propionibacteria (which are typically found on adult skin) were identified in milk (30). Indeed, bacteria are often transferred from one habitat to another within an individual (31). Nevertheless, in the case of human milk further routes may come into play.

Ultrasound imaging has demonstrated the retrograde flow of milk from the infant's mouth back into the mammary gland (32) during suckling. This retrograde flow is made possible by intermittent widening of the milk ducts and could easily explain how *Streptococcus* species, which are commonly found in the infant oral cavity (33), are also commonly found in human milk (22, 26). Strong support for the retrograde inoculation of human milk comes from the previously mentioned CHILD cohort which examined the milk microbiome at 3–4 mo of lactation using 16S rRNA sequencing (28). The most convincingly identified factor influencing milk bacteria was the mode of feeding. If mothers partially fed pumped human milk to their infants in the past 2 wk, within-subject diversity was lower, between-subject diversity was higher, and *Enterobacteriaceae*,*Enterococcaceae*, and potentially pathogenic *Pseudomonadaceae* were found more frequently (28). Thus, one could hypothesize that infant oral cavity bacteria and bacteria present on the breast pump influence the human milk microbiome. The influence of the breast pump would be in agreement with the observation that Pseudomonas were absent in manually expressed milk samples but present in almost half of the pump-collected samples of mothers with mastitis symptoms (34). Furthermore, in preterm infants, it was found that the milk microbiome changed when tube feeding the milk of the mother (eventually combined with donated human milk or formula) was replaced by direct breastfeeding (35). The diversity of the human milk microbiome increased and *Streptococcus* and *Rothia* started to dominate, whereas the infants’ fecal microbiomes became more typical for exclusively breastfed term infants by showing high percentages of Bifidobacteria and low abundances of *Pseudomonas* (35). In addition, Williams et al. (2019) reported very similar microbial communities in human milk and infant oral samples and strong canonical correlations between these niches (36).

Conversely, detailed examination of human milk and maternal skin has revealed that Bifidobacteria and Lactobacilli identified in human milk are not present on maternal areolar skin (37). Thus, maternal skin cannot be the only source of human milk bacteria. Considering also that Bifidobacteria are strictly anaerobic bacteria, makes it highly unlikely that they live on the skin. As such, an enteromammary pathway has been proposed as an additional route. This route requires that certain bacteria are actively transferred into milk from the mother's GI tract (38). An observation supporting this concept is the movement of B-lymphocytes from the maternal intestine to the mammary gland, where they transform and produce secretory IgA, protecting the infant from pathogens to which the mother was exposed (39).

Another interesting finding is that human mammary tissue is not sterile, but contains (independent of lactation) a wide variety of bacteria including *Bacillus*, *Acinetobacter*, *Enterobacteriaceae*, *Pseudomonas*, *Staphylococcus*, *Comamonadaceae*, *Gammaproteobacteria*, *Prevotella*, and *Propionibacterium* (40).

Observations in pregnant mice and humans indicate that bacteria from the maternal digestive tract can spread to extradigestive tract locations (41, 42). Although some questions remain, several studies provide mechanistic details for an enteromammary transfer of viable bacteria in pregnant and lactating women. For instance, there is evidence that dendritic cells can penetrate the GI epithelium and bind nonpathogenic bacteria from the GI lumen (43). Dendritic cells can also open tight junctions between epithelial cells, extend their dendrites into the GI lumen, and collect bacteria while preserving the integrity of the epithelial barrier by producing tight-junction proteins (44). Further, CD18+ cells, including macrophages, may also contribute to this process (45). Once associated with dendritic cells, the bacteria can be transported to other body locations, with lymphocytes exchanging within the mucosa-associated lymphoid system (43). Stimulated cells move from the intestine to other mucosal sites, including the respiratory and genitourinary tracts, salivary and lachrymal glands, and the mammary gland (46). In addition, findings from in vitro and in vivo studies support the proposed enteromammary translocation. Using a cell culture system, a *Lactobacillus gasseri* strain isolated from human milk was found to bind dendritic cells and translocate across a Caco-2 cell monolayer (2). Translocation of bacteria from the GI tract to the mammary gland and into milk was also demonstrated by feeding genetically modified, and thus identifiable, human milk-derived bacteria to mice during pregnancy and detecting these bacteria in their milk (2, 42). In addition, Williams and colleagues provided convincing evidence that, although the microbial taxa in maternal feces and milk are different, ecological variation between these 2 niches are highly correlated (36).

Clinical trials aimed at the treatment or prevention, respectively, of mastitis included the oral administration of specific Lactobacilli strains, isolated from human milk, to lactating mothers. Identification of these strains in the milk of the mothers provides additional evidence for translocation of microbes from the intestine to the mammary gland (47–49).

**FIGURE 1:**
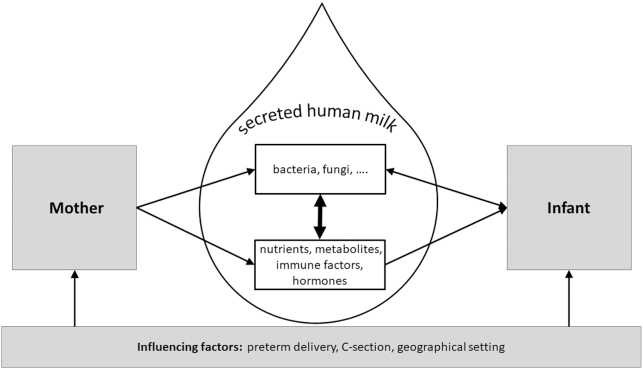
Human milk composition and the human milk microbiome are determined by a complex interplay of biological processes occurring in mother and infant. Besides biological factors such as health and diet of the mother and growth of the infant, the geographical setting, which might include among other factors climate, available economic resources, culture and societal support could influence human milk composition and the microbiome.

Taken together, these findings strongly suggest that, in addition to those bacteria that can be considered to be derived from the skin or the infant's mouth, human milk also likely contains bacteria that are translocated from the maternal GI tract to the mammary gland (**Figure 1**). As such, human milk could contribute to the education of the neonatal immune system, which ultimately enables the infant to differentiate between pathogens and commensal bacteria (46).

## Mode of Sample Collection

The quantitative and/or qualitative content of many human milk components is influenced by a wide range of factors, including genetics, geography, maternal diet, timing (foremilk, hindmilk, and time of the day), gestational age, lactation stage, maternal health, and more (50). This applies to macronutrients, micronutrients, and compounds such as immunoglobulins, cytokines, chemokines, hormones, growth factors, peptides, and oligosaccharides (51–56). The microbial profile of milk seems more variable than nutrients (22), but less is known about factors influencing microbial variation. As for the other milk components, the mode of sampling (e.g. aseptic compared with nonaseptic, full compared with partial breast expression) is likely important. For instance, whether milk is collected aseptically mainly seems to affect *Acinetobacter* species, which represented 32% of total bacteria after sampling without precautions, but were below 2% if precautions were taken to avoid the introduction of bacteria into the milk during the manual collection procedure (57). Overall, strict aseptic collection reduced the bacterial load (estimated by qPCR) by ∼90%; nonetheless, *Streptococcus* and *Staphylococcus* remained the most abundant genera. A high intersubject variability was observed with both sampling techniques. Although aseptic sampling may enable more accurate determination of milk microbes, findings using less aseptic conditions may better reflect the reality experienced by the nursed infant (57). To date, nothing is known about other factors related to sampling specifics (e.g. time of day, fore- compared with hindmilk, time since last feed, fasting compared with fed) in terms of whether they influence bacterial communities in milk. These methodologic issues must be resolved prior to additional research in this area.

## Time Postpartum

The composition of human milk changes with the duration of lactation and may even adapt to the needs of the infant; thus, the question may be asked whether this also applies to the human milk microbiome. In their study, Hunt et al. collected 3 samples each from 16 women at ∼10-d intervals (26). After the breast was cleaned with iodine swabs, a full breast expression was collected using a sterile collection kit, and culture-independent pyrosequencing was applied to describe the microbiota. The results indicated that the stability of the microbial communities differed widely among subjects. Some women showed rather similar patterns, whereas for other women (6/16) no clustering of the samples from the same women was observed (26). Only between 4 to 20% of the operational taxonomic units (OTU) identified in an individual woman were present in all her samples, although those that did represented the majority (60–99%) of the total bacterial abundance (26).

Using similar collection procedures and analytical techniques, Cabrera-Rubio et al. compared the microbiota of colostrum and mature milk collected 1 and 6 mo postpartum (3). In colostrum, the dominating bacteria were the Firmicutes genera *Weissella* and *Leuconostoc* (Lactobacillales), followed by *Staphylococcus*, *Streptococcus*, and *Lactococcus*. These genera were also abundant in the samples collected at the later stage, but *Veillonella* (phylum Firmicutes), *Leptotrichia* (Fusobacteria), *Prevotella* (Bacteroidetes), and TM7 phylum genera had increased. As these are typical members of the oral cavity microbiome (58, 59), this agrees with the possible contribution of the infant mouth microbiome to the human milk bacteria. As time postpartum increased, the human milk microbiome diversity decreased, as indicated by differences in the rarefaction curves, which estimated a higher total number of OTUs from the colostrum samples compared with more mature samples (3). Furthermore, principal component analysis clearly differentiated the colostrum microbiome from more mature samples, indicating as for other milk components, a change of the microbiome with duration of lactation (60).

However, not all studies support substantial changes in the milk microbiome over time. For instance, 21 US women provided milk samples at 9 different time points starting at day 2 after delivery until 6 mo postpartum (61). Relative abundances of bacterial taxa were characterized using a 2-step PCR procedure of bacterial 16S RNA and sequencing based on an Illumina protocol (61). Excluding samples from mothers with reported recent antibiotic intake, results indicated that Firmicutes were most abundant across all studied time points, although the abundance ranged from 23% to 98%. The abundances of the phyla Actinobacteria (range 0.1% to 71%), Proteobacteria (0.1% to 21%), and Bacteroidetes (0.1% to 27%) were mostly considerably lower (61). There was no overall association of time postpartum with abundance of any phylum, although there was a time effect on the genera *Veillonella*,*Granulicatella* (both Firmicutes), *Propionibacterium* (Actinobacteria), and *Prevotella* (Bacteriodetes). Considering the differences of the study populations and methods applied, these findings agree in general with other studies, but in addition may indicate that it is mostly the rare genera that change with time whereas the major genera are more stable. The absence of statistically significant time trends over a 6-mo period with 9 studied time points indicates that time postpartum has minimal (if any) influence on the milk microbiome compared to random variation with time and differences between mothers. It is important to note that not all studies to date have applied adequate statistical tests to their findings, thus making them difficult to interpret. Future studies should utilize adequate statistics to determine whether purported changes (often noted looking at graphical illustrations, such as stacked bar charts) in bacterial community structure over time are actually statistically significant.

## Maternal BMI

Some emphasis has been put on investigating associations between maternal obesity and the human milk microbiome. This effort has largely been based on findings that human milk components such as the hormones leptin and insulin, fatty acids, and some cytokines are associated with BMI (62–67). In addition, obese humans often have a less diverse GI microbiome than healthy weight individuals (68), and fecal microbiomes of pregnant women may be affected by obesity (69). Similarly, some studies have suggested that obese mothers have a less diverse human milk microbiota composition compared with healthy weight mothers (3). Correlation analyses revealed that maternal prepregnancy BMI was positively associated with colostrum counts of *Lactobacillus* and *Staphylococcus*, but negatively with *Bifidobacterium* counts in milk samples in infants aged 6 mo. These findings were confirmed by mixed model analyses, which also found an increase in the number of total bacteria in the milk with increasing maternal BMI. Similarly, an association between gestational weight gain and human milk microbiota was reported, with the microbiome being less diverse with increased weight gain (3). Extending the examinations in the same Finnish cohort, it was found that Bifidobacteria were lower and Staphylococci higher in overweight women than in healthy weight women during the first month of breastfeeding, whereas both transforming growth factor (TGF)-β2 and soluble CD14 tended to be lower in the overweight women (70). The complexity of the interaction between human milk cytokines and microbiota was obvious from the association differences between healthy weight and obese women. Concentrations of soluble CD14 were positively related to *Bifidobacterium* abundance, and concentrations of IL-10 and IL-4 were negatively associated with the abundance of *Akkermansia muciniphila* in healthy weight mothers, whereas this was not the case in obese mothers (70).

## Maternal Health

Additional correlates of maternal health have also been found to be related to variation in the human milk microbiota. For example, using selected primers for Bifidobacteria, Grönlund and colleagues showed that in the milk of allergic mothers (*n* = 52) the counts of Bifidobacteria were significantly lower than in the milk of nonallergic mothers (*n* = 8) (71). This corresponded to cytokine differences between healthy mothers and those with atopic diseases. After controlling for covariates with a strong influence (e.g. smoking, season of birth, and urogenital infections), maternal asthma was associated with increased concentrations of IL-5 in human milk collected 2 wk after delivery (72). In the same study, including samples from 115 women, maternal rhinitis was linked to lower concentrations of IL-5 and IFN-γ in milk, whereas maternal eczema was associated with decreased concentrations of IL-6 (72).

Milk produced by women with celiac disease contained not only lower concentrations of secretory IgA, TGF-β1, IFN-γ, and IL-12, but also lower relative levels of *Bifidobacterium* and *Bacteroides* (73). However, it is important to note that this does not imply causality, and that diets of women with celiac disease vary drastically from those without disease. In milk samples of HIV-positive mothers from Africa, a higher bacterial diversity and more *Lactobacillus* spp. were detected compared with noninfected women (76% compared with 36%) using culture techniques (74). Again, causality and directionality cannot be assessed from these sorts of studies, and it is as likely that microbiomes are changing in response to the disease than vice versa.

A case report from a woman undergoing chemotherapy for Hodgkin's lymphoma during pregnancy and lactation showed, based on a principle component analysis, that the milk bacterial profile and its diversity differed before and after chemotherapy (40). Interestingly, especially Bifidobacteria, Eubacteria, and Lactobacilli, which are considered beneficial for the infant, decreased with chemotherapy (40). This suggests that consideration also needs to be given to drugs, other than antibiotics, administered to lactating women with regard to a potential influence on the milk microbiome (40).

The observation that chemotherapy may influence the human milk microbiome and presumably also the mammary microbiome, raises the question about the mammary tissue microbiome, which might influence the milk microbiome. This has not been studied intensively, but a tendency towards a lower number of OTUs in tumor tissue compared with healthy tissue has been shown (75). However, only 11 of 1614 OTUs were significantly different, with the *Methylobacterium* species being more abundant in tumor tissue and the *Sphingomonas* species more abundant in paired normal tissue (75). Interestingly, an inverse association between the advancement of the disease and the bacterial load (determined by qPCR) in tumor tissue was observed, but not in paired normal mammary tissue. This raises the possibility of an interaction between microbial composition and growth of malignant tissue, but in no way provides causal evidence (75).

## Mode of Delivery

Mode of delivery has been reported to be associated with variation in the microbiota across multiple body habitats of the offspring and longer-term correlates in the GI microbiome have been observed (13). It is widely accepted that natural vaginal delivery and cesarean section (C-section) influence the infant fecal microbiome differently, which may contribute to the long-term consequences of the mode of delivery, including an increased risk of obesity (76) and diseases related to immune disorders (1). Nevertheless, the question as to whether the human milk microbiome of women delivering vaginally differs significantly from the microbiome of those delivering by C-section remains unanswered. In one of the first investigations of this topic, qPCR was used to estimate bacterial abundance in colostrum (days 1–5), transitional milk (days 6–15) and mature milk (day 17) collected from 13 Spanish women (77). Results indicated that in colostrum and transitional milk, the total number of bacterial gene copies was higher after C-section than after vaginal delivery, but no significant differences in the examined genera (*Streptococcus*,*Staphylococcus*,*Lactobacillus*,*Bifidobacterium*,*Enterococcus*) could be found (77). A study of a group of 18 Finnish mothers indicated decreased amounts of *Leuconostocaceae* and increased *Carnobacteriaceae* after elective C-section compared with vaginal delivery in colostrum and mature milk samples (3). An indication of the importance of physiological processes during delivery for the milk microbiome to be established may come from the observation that samples of mothers with nonelective C-sections were more similar to those of mothers with vaginal delivery than the samples obtained after elective C-sections (3). Using similar techniques to analyze milk samples collected from 10 Spanish mothers at 1 mo postpartum, a higher diversity (500 species-level OTUs) was reported for milk samples of mothers with vaginal delivery compared with only 250 OTUs in mothers giving birth by C-section. A principal component analysis of the bacterial composition (species level) clearly separated the milk microbiome of mothers with vaginal delivery from those undergoing C-section. The microbial profiles indicated a trend toward a higher relative abundance of *Staphylococcus* (*P* = 0.085) after C-section compared with after vaginal delivery (60). However, subsequent studies performed in Canada (39 women, 1 sample per subject, day 6 or later postpartum), China (70 women, 3 samples per subject, days 0–4, days 5–11, and 1–2 mo postpartum), and the USA (107 mother-infant pairs, 0 to 365 d postpartum) did not find significant influences of the mode of delivery on the milk microbiome (57, 78, 79). In a study aiming to elucidate the associations between milk fatty acid composition and milk microbes, milk samples were collected at one mo postpartum in Spain, Finland, South Africa, and China; in each location, the researchers studied 10 women with vaginal delivery and 10 women with C-section (80). Results suggested that the mode of delivery is associated with variation in the milk microbiome, but the associations are country specific. In a very recent Finnish study the effect of intrapartum antibiotics in vaginal and C-section deliveries on the human milk microbiome one mo after delivery were studied (81). The study confirmed an effect of the mode of delivery on the milk microbiome and found an independent, albeit modest, effect of intrapartum antibiotics. Nevertheless, of importance may be that species of the genus *Bifidobacterium* were only found in the milk of mothers without antibiotics (81). Thus, additional factors may explain some of the contradictory findings of the studies (80), and again suggest that what is normal in one region and society may not be typical in another.

Going beyond the determination of the abundance of specific genera in an Italian study including 29 women, the colostrum (days 0–3) microbiome was analyzed and the bacterial network described using mathematical modeling (Autocontractive Map, an unsupervised artificial neural network) (82). Bacterial diversity seemed higher in milk produced by women delivering vaginally compared with those delivering by C-section. Higher counts of *Streptococcus* and lower counts of *Staphylococcus* and *Pseudomonas* were detected in milk of women delivering vaginally compared with those delivering by C-section. Interestingly, there was a higher contribution of environmental bacteria in the C-section group (82). Besides the abundance of some bacterial genera, interactions between microorganisms, as described by the mathematical model, were also significantly different between C-section and vaginal delivery colostrum. This is intriguing as it may suggest different bacterial interactions in human milk after C-section or vaginal delivery, which could potentially even affect pathogenicity or beneficial activities, respectively, of some of the present bacteria (82). Additional work at the strain level as well as metatranscriptomics will be needed to explore this possibility.

## Secretor Status

In contrast to bovine milk which has low concentrations of oligosaccharides, human colostrum and mature milk typically contains >20 g/L and 12–15 g/L HMO, respectively (83). There are a myriad of HMO molecules, and mothers can be grouped according to the genetically determined secretor status. Secretors express α1–2-fucosyltransferase (the product of the *FUT2* gene); roughly, 4 g/L of 2’FL (2’-*O*-fucosyllactose) is present in their milk, whereas the milk of nonsecretor mothers contains hardly any 2’FL (84). Together with the Lewis gene, which encodes for α1–3/4-fucosyltransferase (*FUT3* gene), 4 human milk groups are defined according to HMO concentrations and composition (85). The secretor groups differ not only in the content of fucosylated HMO and total concentrations of HMO, but also in the relative contributions of neutral and acidic (sialic acid-containing) HMO. These differences may affect the infant GI microbiome (84–86). Results from a study in California, including 44 mother-infant dyads with human milk and infant stool samples collected at 6, 21, 71, and/or 120 d postpartum, indicated that Bifidobacteria are established earlier and more often in infants of secretor mothers than in infants of nonsecretor mothers (86). As some strains of Bifidobacteria can metabolize HMO and use them as an energy source, these results are not unexpected and agree with the observation that infant feces with high levels of Bifidobacteria contains fewer fecal HMOs and more fecal lactate as HMOs are consumed and lactate is produced by Bifidobacteria (86).

A pilot study in Australian children supported these findings, indicating that microbiomes found in stool samples collected during the third year of life from children of nonsecretor mothers contain more *Prevotella*,*Phascolarctobacterium*, and *Ruminococcaceae* (87). Consequently, the child's and mother's secretor status may have a long-term impact on the child's GI microbiome beyond that seen during the breastfeeding period, implying some kind of early programming which may impact long-term health (87). Considering the potentially important effect of HMOs on the infant GI microbiome, it is interesting to investigate potential interactions of HMOs and milk microbes with regard to the infant GI microbiome. Indeed, the analysis of HMOs and microbiota composition in 11 colostrum samples showed that higher total HMO concentrations were associated with higher counts of Bifidobacteria (88). Detailed investigations on the species level revealed a positive correlation between sialylated HMOs and *Bifidobacterium breve* and nonfucosylated/nonsialylated HMOs and *Bifidobacterium longum*. A further positive correlation was observed between fucosylated/sialylated HMOs and *Staphylococcus aureus*. In a study with Spanish mothers, where HMOs and microbiota were determined in colostrum, transitional milk, and mature milk, mainly *Lactobacillus*, *Staphylococcus*, and *Streptococcus* species were associated with HMOs (89). In the CHILD cohort, associations between fatty acids and HMOs in milk with the human milk microbiota were studied (90). The findings support the importance of HMO, as secretor status seems to modify associations of some milk components with the milk microbiota (90). The results underscore the importance of HMOs, which affect human milk microbes, and together they contribute to the inoculation of the infant GI tract and jointly influence the composition of the infant GI microbiota (88).

## Maternal Diet

Although HMOs are not thought to be influenced directly by maternal diet, other milk components are (91). Thus, associations between the maternal diet and milk microbiota quantity and quality seem plausible. For instance, variation in milk fatty acids, which are strongly influenced by the maternal diet (92), might impact milk microbes either directly or indirectly through effects on the mother's GI microbiome. In this context it is interesting to note the findings of the multinational study by Kumar et al. (80). *Staphylococcus* and several other genera seemed negatively correlated with MUFA percentages in milk triglycerides and the genus *Streptococcus* was negatively correlated with the relative abundance of SFAs in milk triglycerides. *Bifidobacterium* and *Lactobacillus* genera showed negative associations with MUFAs and n–3 PUFAs in milk phospholipids (80). Associations differ between milk triglyceride fatty acids, which form the core of the milk fat globules and contribute >98% of the fatty acids to milk fat, and phospholipid fatty acids, which originate from the milk fat globule membrane trilayer. However, corresponding associations between dietary fatty acids and maternal fecal bacteria were not observed in a study of US women (93). Taken together, this indicates that the associations are complex and relations between dietary fatty acids and the milk microbiome should be investigated in the context of other biological and environmental factors. A study of the associations between the milk metabolomic profiles and the milk microbiota in the same subjects also revealed complex relations, which might be obscured by many further factors (94).

In the most detailed study to date relating maternal diet to the milk microbiome, Williams and colleagues obtained dietary intake data via 24-h recalls at 2, 5, and 10 d and 1, 2, 3, 4, 5, and 6 mo postpartum and linked these data to the milk microbiome data obtained at the same time points from 21 lactating women in the USA (61). Few associations were found between dietary intake variables and relative bacterial abundance in milk within each time point. However, after averaging intake and microbiome data over the full observational period, a myriad of significant associations of phyla or genera, respectively, with specific nutrients (e.g. negative association between Corynebacteria and SFAs as well as MUFAs) and macronutrients (e.g. inverse association between total carbohydrate intake and milk Firmicutes) were noted (61). The emergence of these associations after averaging data over the time points is noteworthy and likely reflects the fact that chronic nutrient intake is more important than acute nutrient intake in shaping the GI microbiome (61). The authors posit several possible mechanisms whereby maternal diet could influence the milk microbiome. For instance, it is possible that (probiotic) bacteria in the diet could reach the mammary gland and be integrated into milk. In addition, micro- and macronutrient intake could influence the composition of bacteria residing in the maternal GI tract and these may reach the mammary gland via the enteromammary pathway. Alternatively, different bacteria may reside in the mammary gland via altered micronutrient content in that microenvironment. This possibility is indicated by findings in a nonhuman primate model, in which 40 cynomolgus macaques were randomized for 31 mo to a “Western diet” providing high intakes of animal protein, saturated fat, and sodium, but low in monounsaturated fat and n–3 fatty acids or a “Mediterranean diet” with high amounts of MUFAs, mainly plant-derived protein, but some protein from fish and dairy, and low in refined sugar (95). Examination of mammary tissue samples indicated effects of the diet on the mammary-gland microbiome and metabolome with 10 times higher *Lactobacillus* abundance and higher bile acid and other bacteria-derived metabolites, respectively, after the Mediterranean diet (95). Furthermore, metabolic products (including vitamins and other nutrients) of one species may influence the growth of other species; this could also, at least in part, explain correlations among the abundances of different genera in the milk microbiome (96).

## Geographic Setting

It is noteworthy that many aspects of the milk microbiome (e.g. diversity, composition) differ among studies conducted in different geographical settings. This can be partially explained by different analytical methods (16), but other factors related to culture and behavioral traditions, including diet, hygiene, and risk of parasites, are likely also relevant (97). Moreover, it is possible that variation in other milk components (e.g. vitamins, immune factors) might affect microbial growth in the mammary gland. Significant differences have been shown among the human milk immune composition of samples collected in Russia, Italy, and the UK, even with adjustment for parity, maternal age, maternal atopy, mode of delivery, living conditions, exposure to tobacco smoking, and frequency of fish, fruit, or probiotic intake (98). The reported and other unidentified factors may contribute to the differences observed among the milk microbiomes studied in different geographical settings (80).

The most comprehensive study on the influence of the geographic setting on human milk composition and microbiome so far is the INSPIRE study. In this study, 370 milk samples from 10 different collection sites, including European and rural African sites, were analyzed for 23 different immune factors using a multiplex immunoassay (55). The authors identified only IgA, IgG, IgM, TGF β2, epidermal growth factor, and the chemokines CXCL1 (Groα), IL-7, IL-8, and CCL4 (MIP1β) as core soluble immune factors detectable in all samples (55). The INSPIRE study also examined HMO concentrations and found striking differences among the sites (56). Among the 19 HMOs analyzed, only 5 HMOs did not show significant differences between collection sites. Conversely, the rest showed substantial differences among countries, with 3-fucosyllactose and disialyllacto-*N*-tetraose being >4 times higher in milk collected in Sweden than in milk from rural Gambia. There were associations of time postpartum, maternal age, and maternal BMI with concentrations of several HMOs, but this did not fully explain the observed differences among geographical locations (56). The INSPIRE project also provides comprehensive data on the human milk microbiome (29). Although a total of 15 phyla were identified in the worldwide collected milk samples, almost 98% of the identified taxa were contributed by only 4 phyla (Firmicutes, Proteobacteria, Actinobacteria, Bacteroidetes). The INSPIRE finding with the highest clinical relevance might be that the milk microbiome variation is associated with variation of the infant fecal microbiome; and correlation analyses indicated that GI tract Lactobacilli of the infant could be influenced by milk Lactobacilli. With respect to the determinants of the milk microbiome, the INSPIRE study emphasized the complexity as variation between and within the studied cohorts was similar, demonstrating that environmental and individual factors are both important (29). A further focus of the INSPIRE project will also be the investigation of the association of the milk microbiome with HMOs and other major or minor human milk components. Initial analyses showed that the associations are complex, but there is a clear difference between secretor and nonsecretor milk, with stronger associations in the milk of nonsecretor mothers (MK McGuire et al. unpublished data).

A recent study in the Central African Republic in groups of hunter-gatherers and horticulturalists identified season of the year and the suckling infant's social network as determinants of the human milk microbiome (99). This agrees with the finding that social interactions are associated with the GI microbiome in nonhuman primates (100). As the human milk microbiome of the studied Africans differs from findings in affluent countries it is not clear how applicable the findings are for other settings, but they indicate that the social environment should be considered in studies of the human milk microbiome.

## Mastitis

Full breastfeeding for ≥4 to 6 mo is recommended as optimal infant nutrition (101), but breastfeeding rates are substantially lower than this goal. One of the underlying reasons is the occurrence of mastitis, i.e. the inflammation of one or more mammary glands, affecting up to 33% of lactating woman (2, 102) and is a primary reason of decreased milk production, which may lead to early termination of breastfeeding (102, 103). In many countries no standard protocols or routine procedures for microbiological analysis of human milk are in place and a lack of established diagnostic criteria contributes to the wide variation of reported incidence rates (2).

Human milk usually contains between 100 and 1000 colony-forming bacteria/mL of different species, typically *Staphylococcus*,*Streptococcus*,*Corynebacterium*,*Lactobacillus*,*Leuconostoc*,*Bifidobacterium*, and others (2, 104). Mastitis involves a state of dysbiosis, where one or two species dominate and bacterial numbers increase up to one million colony-forming units/mL. Symptoms of classical, acute mastitis include pain during breastfeeding, redness of the breast, and fever. Symptoms are often attributed to toxins produced by *Staphylococcus aureus*, which can reach circulation and thus affect the whole body. In cases of subacute mastitis, only local pain occurs in the breast or less milk is produced. This is typically associated with the appearance of *Staphylococcus epidermis* and *Streptococcus salivarius* in milk. A further variant is granulomatous mastitis, which can cause abscesses. It is associated with *Corynebacterium* and difficult to treat with antibiotics (105). In a Spanish study, milk samples from 1849 women suffering from acute, subacute, or subclinical mastitis were microbiologically analyzed according to a standardized protocol (106). Agglomerative hierarchical clustering separated the samples into 2 groups. The larger group comprised ∼60% of the samples and was characterized by the dominance of *Staphylococcus epidermis*, either alone or in combination with another species. In the remaining 40% of the samples, at least one of the dominating species was a *Streptococcus* species (often from the *salivarius* or *mitis* groups), followed by the second most dominant species, either *Staphylococcus aureus*,*Staphylococcus epidermis*, or a *Rothia* species (106).

Mastitis-causing bacteria are difficult to treat with antibiotics and an alternative treatment counteracting dysbiosis associated with mastitis could be the use of probiotics. (107). In 2 clinical trials, the oral application of Lactobacilli strains, isolated from healthy women, led to a significant drop in counts of pathogenic bacteria, linked to a significant improvement of mastitis symptoms after only 2 wk (47, 48). Interestingly, the supplemented *Lactobacillus salivarius CECT5713* and *Lactobacillus fermentum CECT5716* strains were detected in the milk (48). The effectiveness of probiotics was further supported by a detailed investigation of milk and serum of lactating women with and without mastitis receiving *Lactobacillus salivarius* for 21 d (107, 108). In infected women, probiotic intake led to a reduction in milk bacteria, reduced leukocyte counts, and changes of cytokines in milk and serum, as well as a reduction of some markers of oxidative stress in milk (107). Significant changes in gene expression of milk somatic cells of women with mastitis after the treatment with the probiotic were also detected. Inflammatory and cell-growth related signaling pathways and specific genes were identified as potentially responsive targets of the probiotic treatment (108). The milk macronutrient content was not changed by probiotics, but human milk electrolyte content was normalized, indicating improved integrity of the mammary gland epithelia (107). Urine metabolomics, using NMR and partial least square discriminant analysis, revealed that in women with mastitis, 21 d of probiotic intake induced changes of the metabolome (109). Decreased lactose excretion in the probiotic group indicates normalization of mammary permeability and the observed decrease of ibuprofen and acetaminophen catabolites in urine suggest reduced consumption of pharmaceuticals and thus a clinical benefit of supplementation with the probiotic *Lactobacillus salivarius* (109). Taken together, this indicates that probiotics are a valid alternative to antibiotic treatment (48).

Moreover, the preventive potential of *Lactobacillus salivarius* in relation to mastitis was shown in a double-blind randomized study including 108 women with previous experience of mastitis (49). Women were randomly assigned to consume either a milk powder (placebo) or the probiotic *Lactobacillus* supplement (intervention) between week 30 of pregnancy and delivery. Results showed that not only were bacterial counts in the probiotic group significantly lower, but most importantly, the incidence of mastitis during the first 3 mo of lactation, which is the phase with the highest mastitis risk, was significantly lower in the probiotic (25%) compared with control subjects (57%) (49).

## Preterm Delivery

Another situation potentially impacting the human milk microbiome is preterm birth, defined as birth before 37 completed weeks of gestation, which affects ∼10% of all births (110). As for term infants, the optimal nutrition for preterm infants is the milk of the mother, but fortification with protein and other nutrients is required (111). Among the factors that may lead to differences between the preterm and term microbiome are those which are known to impact microbial colonization of the infant GI tract, such as antibiotic therapy of the mother or infant, invasive medical procedures including C-section, delayed or limited physical contact between mother and infant, delayed enteral feeding, or prolonged stay in neonatal intensive care (112, 113).

Compared with healthy, full-term, vaginally delivered newborns without antibiotic treatment, in preterm infants Lactobacilli and strict anaerobes such as Bifidobacteria and *Bacteroides* are reduced, whereas bacteria associated with hospital environments, such as *Staphylococcus*,*Enterococcus*, and *Enterobacteria*, are increased (114, 115). In a longitudinal study, 45 preterm infants were monitored until the postnatal age of 60 d collecting 2–11 fecal samples per infant, which were analyzed by 16S RNA sequencing to describe bacterial number and diversity (116). The authors found the development of the microbiome to be more related to postmenstrual age than to postnatal age. They identified 4 partially overlapping phases in the early microbiome development in preterm infants: *1*) *Staphylococcus* dominance, *2*) *Enterococcus* dominance, *3*) *Enterobacteriaceae* dominance, and *4*) high abundance of *Bifidobacterium*. In term-born infants usually only the decreasing portion of phase 3 is observed (116). Nevertheless, the data also indicated that with human milk feeding at least the microbiome of moderately preterm-born infants can develop towards a *Bifidobacterium*-dominated microbiome as in term infants (116).

The clinical importance of the intestinal microbiome for preterm infants has been highlighted by the observation that the ratio between Firmicutes and Proteobacteria is different between infants suffering from NEC and nonaffected controls as early as 1 wk before diagnosis (117). Since NEC is life threatening for infants, it highlights the importance of the intestinal microbiome and factors potentially influencing it, including human milk feeding and associated human milk microbes. The establishment and succession of bacterial communities in infants may have a profound impact on their health. Therefore, information about the meconium and the early fecal microbiota is of great importance in hospitalized preterm infants.

Thorough examinations of the microbiome of a group of preterm infants (*n* = 26, born prior to 32 weeks of gestation, with a weight below 1500 g), who were partially followed up until the age of 2 y, revealed a series of interesting details about microbiome development in preterm infants (118–122). The number of antibiotic-resistant high-risk clones was high in the samples collected at early ages, whereas the majority was replaced by less critical clones in the samples of those aged 2 y. The occurrence of *Serratia* in the early age samples seemed to be influenced by variables related to prematurity, such as a gestational age of only 30 wk, longer hospital stay, prolonged antibiotic therapy, and mechanical ventilation. On the other hand, *Escherichia* occurrence was related to a higher birth weight and gestational age, fewer days of antibiotic treatment, and shorter duration of tube feeding. With advancing age, the diversity of the microbiome of the preterm infants (as indicated by the Shannon–Weaver diversity index) increased and was, at the age of 2 y, no longer statistically different from that of term-born infants. Firmicutes contributed ∼63% to total bacteria in the meconium samples, Proteobacteria dominated in fecal samples collected at the age of 21 d (118), and in the samples from those aged 2 y the majority of bacteria again belonged to the Firmicutes phylum (79%), whereas much lower contributions were observed for Actinobacteria (10%), Bacteroidetes (8%), and Proteobacteria (3%) (118).

## Tube Feeding

In relation to the microbial colonization of the preterm infant GI tract, it is important to consider the specific situation of tube feeding of maternal milk, donor milk, or infant formula. During intragastric feeding the milk has to pass through the tubing, while it is at temperatures above 30°C. This procedure can lead to the buildup of biofilms and microbial growth, which may considerably influence the number and composition of the bacteria to which the infant is exposed to (119). Examination of the microbiome after passage through the external tubing, using culture-dependent techniques, revealed a significantly higher diversity in maternal milk (Shannon diversity index 1.2 ± 0.1) compared with the other feeds (0.6 ± 0.1 and 0.4 ± 0.1, stored milk and formula, respectively). *Staphylococcus* was the most frequently (93% of samples) detected genus in the maternal milk samples, *Enterococcus* was the most frequent in donor milk (49%), and in infant formula the highest frequencies (27%) were observed for both *Enterococcus* and *Klebsiella* (119). Although there were differences between the 3 types of feeding, they were more similar than expected, considering that the milk of the donor was pasteurized and infant formula should initially be germ-free, whereas the maternal milk was refrigerated or frozen before feeding. These findings highlight the importance of maintaining the highest hygiene standards, including frequent tubing change to avoid excessive buildup of biofilms during the preparation and enteral application of milk feeds (119).

Interestingly, the situation is even more complex, as nonheated human milk contains a mixture of biologically active compounds which influence microbial growth, such as HMOs, maternal antibodies, lactoferrin, and lysozyme. This complexity of human milk nutrients and its impact on the milk microbiome supports the relevance of improved understanding of the interaction between the milk microbiome and GI microbiome for all infants and especially for preterm infants. The good news is that when preterm infants were monitored until the age of 2 y, results indicate that microbial diversity increased and critical strains typically associated with hospital environments were replaced by bacterial strains found widely in the community (118).

## Regulatory Aspects

Although additional basic research and translational research is required to put the acquired knowledge about the human milk microbiome into practice, prior to the introduction of any new product on the market the regulatory requirements must be met. Scientists should be aware that the constant advancement of science, including changes of what was previously considered a dogma, makes it difficult for regulators to make “quick” decisions. Regulatory bodies are only able to gradually adapt to scientific progress and they depend on close cooperation with scientists who are the forerunners. The complexity of the issue becomes obvious when considering the definitions of pre- and probiotics. As established by groups of scientists, both definitions mention health effects (123, 124), which from the regulatory point of view would require the approval of health claims. Thus, these definitions cannot easily be accepted by regulators. Considering that it took the World Allergy Organization several years to generate position papers on the effectiveness of pre- and probiotics for allergy prevention in children (125, 126), slow decision-making processes in politics and legislation are not surprising.

For the European Union (EU) member states, the European Food Safety Authority (EFSA) scientifically assesses the risks and benefits of novel foods, but the final decisions are made by the European Commission, which also approves corresponding communication. In the EU, since 1997 the novel food regulation (current version EU Regulation 2015/2283) defines the approval process and requires that all available scientific data, in favor or not in favor of a novel food, be presented by the applicant (127). Foods not consumed to a significant degree by humans in the EU before May 1997 are considered a novel food and require approval, which also applies to probiotics (128). The prebiotic oligosaccharides lacto-*N*-neotetraose (LNnT) and 2’FL, which are of interest for infant formulas, have obtained approval as novel food ingredients (129–131). For the approval of the introduction of probiotics, it must be demonstrated that consumption is risk-free. In this context an important document is the qualified presumption of safety (QPS) list of microbes, which have already been assessed by the EFSA Panel on Biological Hazards (132). The list is updated at least annually and demonstrating safety is simplified for bacteria which are already listed.

In addition to safety approval for the target population, for a health claim, causality in relation to positive consequences for disease risk factors must be sufficiently demonstrated (133). Requirements for the approval of health claims are high, as health claims can be rejected due to methodological criticism of the presented clinical data or lack of data referring to the target populations (134). Health claims for HMOs have not been applied for at the time of writing this review.

Rapid translation of scientific advance into practice is crucial and regulatory requirements need to be considered and improved as well along with the increasing knowledge and progressing science. Regulatory issues can be minimized by applying generally accepted methods, existing biomarkers, and risk factors when compiling the documentation for approval of a novel food component.

## Conclusion

**FIGURE 2:**
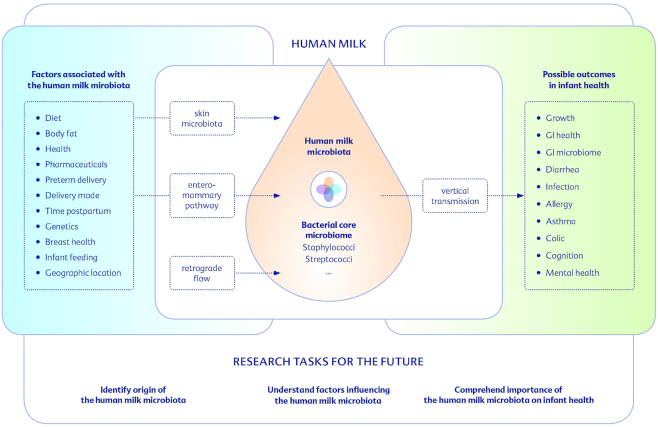
Considerable knowledge in relation to the human milk microbiome has already been acquired but further research on the origin of the human milk microbiota, the influencing factors, and their association with health outcomes is needed to enable consideration of the human milk microbiome in future recommendations and dietary products. GI, gastrointestinal.

The improvement of infant health and development depends on increased understanding of the mechanisms of how human milk components, including HMOs, immunologically active compounds, and microbes, interact with maternal nutrition, health, environment, lifestyle, and most importantly with infant health. Understanding of the human milk microbiome, including its determinants, has progressed fast and the available findings strongly support the hypothesis that the milk microbiome is an important component of early postnatal development (**Figure 2**). Further research and specifically clinical intervention studies, focusing on the human milk microbiome, are needed to improve recommendations for lactating mothers, clinical practice, and the introduction of modified and optimized dietary products for mothers-to-be and/or lactating women and their infants.

## Notes

The workshop on which this manuscript is based was supported by an unrestricted educational grant provided by HiPP GmbH & Co. Vertrieb, Pfaffenhofen, Germany.

Author disclosures: With the exception of the financial support for workshop participation and manuscript preparation by HiPP GmbH & Co. Vertrieb, Pfaffenhofen, Germany, the authors report no conflicts of interest.

Abbreviations used: C-section, cesarean section; EFSA, European Food Safety Authority; GI, gastrointestinal; HMO, human milk oligosaccharide; NEC, necrotizing enterocolitis; OTU, operational taxonomic unit; TGF, transforming growth factor; 2’FL, 2’-*O*-fucosyllactose.
